# Institutional contexts structure wellbeing-relevant psychosocial opportunities in residential disability services: a national documentary analysis in Romania

**DOI:** 10.3389/fpsyg.2026.1968704

**Published:** 2026-10-12

**Authors:** Sorina Corman

**Affiliations:** Community Development Lab, Lucian Blaga University of Sibiu, Sibiu, Romania

**Keywords:** agency, belonging, community participation, deinstitutionalization, positive psychology, residential disability services, self-determination, wellbeing

## Abstract

Positive psychology increasingly recognizes that wellbeing depends on meaningful social and organizational contexts, yet residential disability services are rarely examined as environments that structure the practical opportunities through which autonomy, competence, relatedness, agency, and social inclusion can develop. We conducted a directed documentary content analysis of the complete eligible 2025 corpus of official Romanian monitoring reports on residential services for adults with disabilities: 54 reports representing 67 services. A 31-code framework covered agency, belonging, capability development, community participation, and institutional mechanisms. Each report-code combination was classified as Enabling, Restrictive, Mixed, or Not reported, with original-page verification, negative-case analysis, an exhaustive second consistency pass, and sensitivity analyses for cluster and thematic reports. Across 1,674 report-code combinations, 1,410 contained directional evidence: 334 Enabling, 560 Restrictive, and 516 Mixed. Freedom of movement was the clearest structural discriminator. Formal procedures frequently exceeded evidence of usable agency; positive staff relationships coexisted with structural restrictions; organized community outings were more common than independent mobility or reciprocal membership; education and employment opportunities were limited; and administrative relocation was not equivalent to resident-led deinstitutionalization. Small scale and community location were therefore insufficient by themselves. Residential services functioned as psychosocial opportunity structures: wellbeing-relevant conditions depended less on service labels than on whether organizational resources were converted into resident-controlled opportunities for choice, movement, relationships, competence, valued social roles, and community living.

## 1 Introduction

A growing contextual strand in positive psychology examines not only individual strengths and psychological resources but also the social and organizational conditions that make positive functioning possible. This emphasis is directly relevant to meaningful contexts: organizations can provide resources for agency, belonging, participation, and quality of life, while rigid practices and structural constraints can narrow those same opportunities. Self-determination theory (SDT) offers a psychological foundation for this view because autonomy, competence, and relatedness are basic needs whose satisfaction depends partly on the surrounding social environment rather than on individual disposition alone (Ryan and Deci, 2000). For adults who rely on residential support, the service is therefore not merely a location of care. It is an everyday organizational and social context in which rules, relationships, staff practices, physical design, decision arrangements, and community connections shape what residents can understand, choose, do, and sustain.

This contextual interpretation is consistent with research showing that organizational characteristics matter in disability services. Organizational climate has been associated with staff satisfaction and burnout, and residential models differ in organizational conditions that can influence support practice (Chou et al., 2010; Thompson and Rose, 2011). Research in intellectual-disability services further indicates that organizational values, resources, staff stability, and the living environment shape the quality of support and resident experience (Olivier-Pijpers et al., 2019, 2020). Moos (2012)) similarly conceptualizes residential environments through institutional context, physical features, policies and services, resident and staff characteristics, and social climate. More recent work on organizational readiness for change highlights leadership, resources, and organizational dynamics as conditions affecting whether improvements can be implemented in disability care (Granberg et al., 2025). Together, this literature supports treating residential services as part of the psychological ecology of wellbeing rather than as neutral containers for individual functioning.

A central problem is the distinction between the formal presence of a right, procedure, resource, or activity and the resident's real opportunity to use it. The capability approach is useful here because disadvantage is evaluated in terms of substantive opportunities and functionings, not merely the nominal availability of resources (Terzi, 2020). In the present study, this distinction is linked to SDT, agency, and supported decision-making. Supported decision-making is intended to preserve personal autonomy by enabling a person to make their own decisions with appropriate assistance rather than defaulting to substitute decision-making (Davidson et al., 2015). A complaints procedure, an individual plan, a signed consent form, or a nearby bus route may exist administratively while remaining psychologically or practically unusable if information is not understood, choices are substituted, movement is restricted, or individualized support is absent. We use psychosocial opportunity structure as an analytic description of these environmentally organized possibilities for action, connection, competence, and participation; it is not a psychometric measure of subjective wellbeing.

Belonging and community inclusion require the same distinction between nominal provision and lived opportunity. Relatedness is not exhausted by the absence of conflict or by positive staff interactions: for residents of long-term services it may involve continuity with family, reciprocal peer and intimate relationships, private communication, and roles extending beyond the service. Physical presence in the community is likewise different from community membership. Research on intellectual and developmental disability explicitly distinguishes physical presence from genuine social inclusion, reciprocal relationships, and participation (Amado et al., 2013). An umbrella review similarly found that stronger participation depends on purposeful connections to meaningful opportunities, ongoing support, and community conditions that permit genuine choice (Giummarra et al., 2022). International evidence indicates that movement from institutional to community settings is generally associated with improved quality of life, greater freedom, decision-making, family contact, and social integration, while outcomes remain heterogeneous (Walsh et al., 2010; McCarron et al., 2019). Recent longitudinal evidence further suggests that relocation alone is insufficient: quality-of-life improvements depend on continuing opportunities for choice and decision-making (Navas et al., 2025). This makes it important to distinguish nearby resources, organized outings, independent mobility, and sustained reciprocal roles rather than treating them as interchangeable indicators of inclusion.

Person-centered planning and risk governance provide two further tests of whether organizational context becomes supportive in practice. Person-centered planning is intended to connect preferences and aspirations to concrete support, but changing planning formats without changing power relations, resources, staff practice, and organizational conditions may have limited effects (Mansell and Beadle-Brown, 2004). A systematic review found benefits for choice and community participation but uneven effects on broader outcomes such as employment (Ratti et al., 2016). Protection can similarly support wellbeing, yet blanket restrictions can remove opportunities for autonomy and learning. The dignity-of-risk perspective emphasizes proportionate support for foreseeable risk rather than generalized paternalism (Chicoine and Kirschner, 2022). Locked gates, universal accompaniment, permission systems, surveillance, and low expectations can therefore be interpreted as institutional mechanisms that affect wellbeing-relevant opportunities, not merely as administrative features.

Although previous research establishes that organizational environments, community living, and person-centered support matter, less is known about the mechanisms through which formal rights, resources, and service arrangements are converted—or fail to be converted—into practically usable opportunities across a complete public monitoring corpus. Monitoring reports provide a distinctive empirical window because a single document can juxtapose formal procedures, case-file evidence, environmental observations, staff and resident accounts recorded by monitors, and corrective recommendations. They are therefore suited to tracing institutional processes that may be missed by service labels or formal policy descriptions, while remaining unsuitable for measuring residents' subjective wellbeing directly. The present study analyzes the complete eligible 2025 corpus of Romanian residential disability-service monitoring reports to examine residential services as meaningful organizational and social contexts for wellbeing-relevant psychosocial opportunity. It addresses three research questions: (1) How are enabling, restrictive, and mixed opportunities for agency, belonging, capability development, and community participation documented across residential services? (2) Which recurring institutional mechanisms and configurations distinguish more enabling or community-bridging contexts from containing contexts? and (3) Under what conditions are formal rights, person-centered procedures, activities, and community resources converted—or not converted—into resident-controlled opportunities relevant to autonomy, competence, relatedness, inclusive participation, and community living?

## 2 Materials and methods

### 2.1 Study design

We conducted a structured documentary content analysis of official monitoring reports concerning residential services for adults with disabilities in Romania. The design combined a directed qualitative content-analysis logic, in which a theoretically informed coding framework is applied systematically to text (Hsieh and Shannon, 2005), with matrix-based cross-case comparison consistent with the Framework Method (Gale et al., 2013). The analysis was designed to examine the psychosocial opportunities and constraints produced by residential environments rather than to estimate clinical effects or subjective wellbeing.

The analytic logic was deliberately mechanism-oriented. A service was not assigned a global quality score, and code frequencies were not interpreted as direct measures of resident wellbeing. Instead, the analysis examined how documented practices, rules, relationships, environmental features, professional capacity, and community connections combined to expand or narrow psychosocial opportunities.

### 2.2 Documentary corpus and eligibility

The source frame was the publicly accessible 2025 monitoring-report archive of the Council for Monitoring the Implementation of the UN Convention on the Rights of Persons with Disabilities (Romanian: Consiliul de Monitorizare a Implementării Conven tiei ONU privind Drepturile Persoanelor cu Dizabilită ti), an autonomous Romanian public authority under parliamentary oversight with a statutory monitoring mandate (Consiliul de Monitorizare a Implementării Convent ´ iei ONU privind Drepturile Persoanelor cu Dizabilităt ´ i, 2026). All entries in the 2025 archive were title-screened. Documents entered full-text eligibility review when the title indicated an adult residential disability service or when eligibility remained ambiguous. Eligibility required that the monitoring visit occurred in 2025, that the service was residential, that it concerned adults with disabilities, and that the document reported an initial monitoring visit rather than a response letter or later implementation follow-up. Visit date, rather than upload date, governed temporal eligibility; consequently, one report uploaded in January 2026 was retained because the monitoring visit occurred in December 2025.

Fifty-six potentially relevant documents entered full-text screening. One mixed child/adult complex report representing five services was excluded because findings concerning the adult service could not be separated reliably from findings concerning child services. One follow-up report concerning one service was separated from the primary corpus because its purpose was to assess implementation of previous recommendations rather than an initial monitoring visit. The final primary corpus therefore comprised 54 reports representing 67 residential services for adults with disabilities. The corpus included care and assistance services (CIA/CIAPAD), habilitation and rehabilitation services (CAbR/CRR/CRRN), and protected-living services (LMP/CPVI). This is a complete eligible corpus of reports within the authority's 2025 archive under the stated criteria; it is not a census of every residential disability service operating in Romania.

Four reports covered multiple services and were treated as report-level clusters. Together, these four documents represented 17 services; the remaining 50 reports each concerned a single service. Cluster reports remained single primary analytical units so that findings shared across a document were not multiplied by the number of services it described. Service-specific evidence was attributed to an individual service only when the source report allowed that attribution. This approach protects report-level frequencies from artificial inflation but can obscure some within-report service variation; that limitation is addressed explicitly below and tested through sensitivity analysis. Figure 1 summarizes corpus identification and analytic allocation.

**Figure 1:**
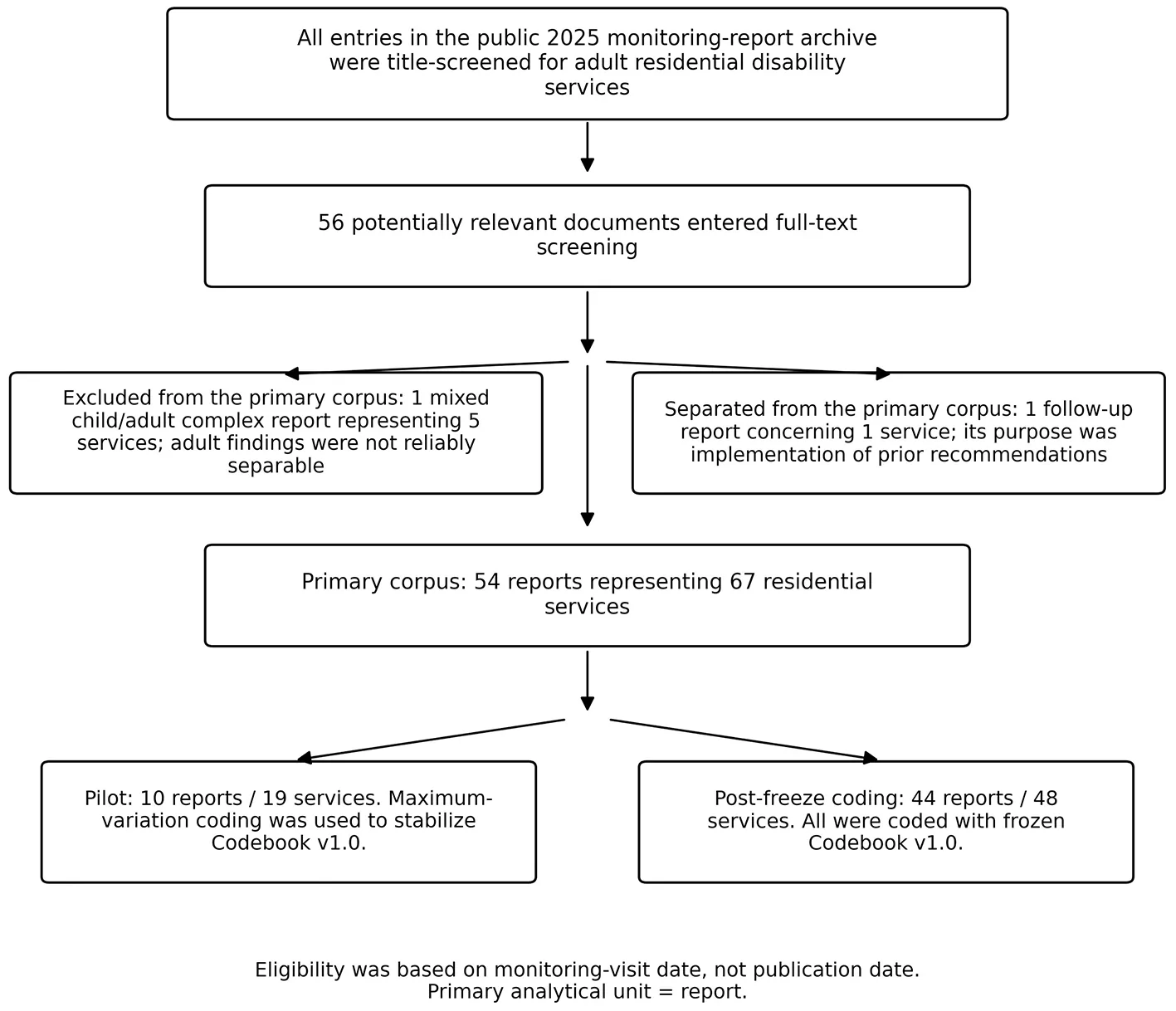
Documentary corpus identification and analytic allocation. The screening unit was the report; service counts are shown where relevant. Eligibility was determined by monitoring-visit date rather than publication date.

### 2.3 Document preparation and evidence extraction

Most source PDFs were image scans and did not contain usable embedded text. Romanian-language optical character recognition (OCR) was therefore used to create page-separated working text for navigation and evidence retrieval. OCR output was never treated as the authoritative source for quotations, dates, numerical claims, or ambiguous wording. High-impact and uncertain findings were checked against visually rendered pages from the original PDF. All source reports were analyzed in Romanian. English evidence summaries and manuscript paraphrases were prepared from the Romanian source text and author-verified against the relevant original page; interpretive decisions were based on the Romanian wording and context rather than on an unverified machine translation.

For each report-code combination, the analytical matrix recorded the report identifier, service type, code, directional classification, source page or page range, type of documentary evidence, a close paraphrase of the evidence, confidence, verification priority, provisional configuration, and an analytic memo. Evidence-source labels distinguished material reported as documentary records, staff accounts, resident accounts, monitoring-team observation, recommendations, or combinations of these sources. These labels characterize what the public monitoring report recorded; the present study did not conduct interviews or observations with residents or staff. Report identifiers and page references were retained specifically to preserve a traceable chain from source document to coded decision.

### 2.4 Codebook and directional coding

The framework was deductive at the domain level and empirically refined at the operational level. Its initial structure drew on Self-Determination Theory, capability and supported-decision-making perspectives, person-centered planning, community participation, and institutional-environment literature. A purposive maximum-variation pilot of 10 reports representing 19 services was then used to test construct boundaries across service types and document structures. Pilot review led to several explicit revisions before full-corpus coding: the former information/decision code was split into accessible information/comprehension (A2a) and supported decision-making/consent (A2b); privacy was separated from intimate/relationship autonomy (A6a/A6b); paternalism and low-expectation narratives were added as E10; and decision rules were tightened for complaints, community proximity vs. actual participation, transition requests, relational warmth vs. structural restriction, and multi-service cluster reports. These changes were documented in a decision log and Codebook v1.0 was then frozen. The remaining 44 primary reports representing 48 services were coded with the frozen 31-code framework, with no silent addition or deletion of constructs during full-corpus application. The complete codebook and decision rules are provided in Supplementary Data 1. The five analytic domains and their code families are summarized in Table 1.

**Table 1:** Analytic domains and code families.

Domain	No. of codes	Analytic focus	Illustrative constructs
Agency	8	Resident influence over ordinary life and major decisions	Everyday choice; comprehension; supported decision-making; money; movement; complaints; privacy; relationships
Belonging	4	Relational continuity and reciprocal connection	Family ties; peer relationships; staff–resident climate; digital connectivity
Capability development	4	Opportunities to develop competencies and future options	Independent-living skills; person-centered planning; education; employment and productive roles
Community participation	5	Conversion of institutional support into ordinary community life	Resource use; independent mobility; organized exposure; community membership; transition
Institutional mechanisms	10	Organizational mechanisms that enable or constrain opportunity	Routine; surveillance; staffing; built environment; medication governance; legal barriers; scale; documentation–practice gap; partnerships; low expectations

Each code was assigned one of four directional classifications: Enabling, Restrictive, Mixed, or Not reported. Enabling indicated documentary evidence that the relevant institutional condition expanded resident opportunity; Restrictive indicated evidence that it narrowed opportunity; Mixed indicated the coexistence of meaningful enabling and restrictive evidence within the same report; and Not reported indicated that the report did not provide sufficient evidence for a directional judgment. Not reported was used conservatively, particularly for thematic reports whose monitoring scope did not cover all domains.

The codebook included explicit boundary rules. For example, the presence of a complaint box did not by itself constitute influence; an empty complaint register was not interpreted as satisfaction; nearby shops or transport were coded as community-bridging resources rather than community participation unless actual use was documented; organized outings were not treated as independent mobility or community membership; and service size was not coded as restrictive unless crowding, over-capacity, loss of privacy, or a resident-level effect of institutional concentration was documented.

### 2.5 Coding workflow and analytical rigor

Coding followed a structured single-analyst workflow supported by generative AI and governed by an explicit audit trail. ChatGPT (version: web application accessed August 2026; model: GPT-5.6 Sol; source: OpenAI, San Francisco, CA, USA) was used for OCR-guided navigation, structured extraction, retrieval of candidate evidence, drafting of close paraphrases, and consistency checks across the report-code matrix. AI output was not treated as an independent coder, an adjudicator, or primary evidence. The author retained responsibility for every directional classification, code-boundary judgment, original-page check, and final adjudication. No subset was independently recoded by a second researcher or subject-matter expert, and the study therefore makes no claim of human intercoder reliability.

The procedure proceeded in four stages. First, the 10-report pilot was coded contrastively to stabilize Codebook v1.0 and its boundary rules. Second, all eligible reports were coded systematically against the frozen 31-code framework. Third, high-impact, ambiguous, and boundary-sensitive decisions were checked against original PDF pages. Fourth, each coding batch underwent an exhaustive second pass after the initial pass to identify over-inference, codebook drift, page-reference problems, and cross-code contradictions before analytical directions were frozen. Any direction or page-reference revision introduced during verification or second-pass review required a written reason in the change log. Contradictory enabling and restrictive evidence was retained as Mixed rather than forced into a single direction, and documentary silence was coded Not reported rather than inferred as absence.

Several safeguards were used to manage interpretive drift and confirmation bias in the absence of a second coder. The codebook was frozen before coding the remaining 44 reports; boundary-sensitive codes were revisited systematically across cases; report-level memos recorded both enabling and restrictive mechanisms; and negative cases were actively sought when an emerging explanation risked becoming too general. For example, small services with restrictive mobility rules, transition-active services with substantial internal controls, and warm relational climates coexisting with surveillance were retained as challenges to simple service-level narratives. Targeted original-page verification, preservation of Mixed evidence, and the batch-specific audit trail provided additional checks. Reporting and methodological transparency were guided by qualitative reporting principles in JARS-Qual and SRQR (Levitt et al., 2018; O'Brien et al., 2014), adapted to a documentary rather than interview-based design.

### 2.6 Cross-case analysis and descriptive counts

After report-level coding was frozen, each case was summarized through its 31-code profile and analytic memo and then compared across cases. Recurrent combinations were described using interpretive families such as enabling/community-bridging, activity-based/mixed, activity-based/containing, and containing/critical. These labels were qualitative summaries of recurring combinations of mechanisms, not latent classes, numerical thresholds, predictions, or service ratings. Each non-thematic report was assigned to one mutually exclusive descriptive family after review of its complete profile; four thematic reports were left uncategorized because their restricted monitoring scope did not support a full configuration judgment. When a profile contained competing features, assignment followed the overall pattern of resident control and mobility considered together with relational support, capability development, community connection, and institutional constraints, while contradictory features were retained in the analytic memo. No numerical cutoff or composite score was used. The labels were used to organize cross-case contrasts rather than to generate inferential statistics.

Counts of Enabling, Restrictive, Mixed, and Not reported decisions were used descriptively to show code coverage and documentary direction. No composite score was calculated for any service. Unless otherwise stated, the quantitative denominator is the report: 54 reports × 31 codes = 1,674 report-code combinations. The figure of 67 services describes corpus coverage and is not treated as 67 independent analytical observations. Cluster reports were counted once at report level; service-specific evidence was preserved only when attributable. Sensitivity analyses excluded the four multi-service cluster reports and/or four thematic reports to test whether principal mechanisms depended on atypical document structures. A mechanism was described as stable only when it recurred across multiple reports and service groups, remained interpretable after deliberate examination of negative or boundary cases, and remained visible in the conservative sensitivity scenario excluding both cluster and thematic reports. This use of “stable” therefore refers to robustness within the documentary corpus, not causal stability or population-level generalizability.

### 2.7 Ethical and data-governance considerations

The study was a secondary documentary analysis of publicly available, de-identified institutional monitoring reports. Although the reports contain human-derived descriptions generated during statutory monitoring, the present research did not recruit, contact, interview, observe, or intervene with residents or staff, did not access non-public case files or identifiable private records, and did not collect new individual-level human-participant data. The unit of analysis was the monitoring report rather than the individual resident. Accordingly, ethics approval and informed consent were not applicable to this documentary analysis.

The derived analytical dataset contains report-level codes, close paraphrases, page references, and audit-trail information rather than resident names, contact details, images, or other direct personal identifiers. Service and report identifiers are retained only to support source traceability. Any documentary excerpt selected for the revised manuscript is checked against the original public page and screened for residual identifiability before use. The source reports were analyzed in Romanian, and English paraphrases or excerpts were author-verified against the Romanian originals. The complete codebook, report-code dataset, decision rules, audit trail, and sensitivity outputs are provided in Supplementary Data 1; exact public report titles and the public archive location are documented to permit source retrieval and corpus reconstruction.

## 3 Results

### 3.1 Corpus coverage and distribution of documentary evidence

The final analysis covered 54 monitoring reports representing 67 residential services. Unless otherwise specified, all directional frequencies use the monitoring report as the analytical denominator; the 67-service figure describes corpus coverage rather than 67 independent observations. Applying 31 codes to each report generated 1,674 report-code combinations. Of these, 1,410 contained sufficient documentary evidence for a directional classification: 334 were Enabling, 560 Restrictive, and 516 Mixed; 264 were Not reported. At domain level, Agency comprised 97 Enabling, 123 Restrictive, 164 Mixed, and 48 Not reported decisions (432 combinations); Belonging, 66/23/76/51 (216); Capability development, 45/107/46/18 (216); Community participation, 75/89/58/48 (270); and Institutional mechanisms, 51/218/172/99 (540). These are documentary directions, not prevalence estimates of resident outcomes or service-quality scores. Full code-level distributions are provided in Supplementary Data 1. The overall corpus and directional coding distribution is summarized in Table 2. Report identifiers used below (C01-C54) map to exact public report titles in the supplementary corpus manifest; examples are concise source-traceable paraphrases rather than quotations.

**Table 2:** Final corpus and directional coding distribution.

Metric	*n*	Interpretation
Primary monitoring reports	54	Primary documentary unit
Residential services represented	67	Includes multi-service report clusters
Report-code combinations	1,674	54 reports × 31 codes
Documented directional decisions	1,410	Enabling + Restrictive + Mixed
Enabling	334	Documented expansion of psychosocial opportunity
Restrictive	560	Documented narrowing of psychosocial opportunity
Mixed	516	Coexisting enabling and restrictive evidence
Not reported	264	Insufficient evidence for a directional judgment

The corpus comprised 30 CIA/CIAPAD reports representing 32 services, 19 CAbR/CRR/CRRN reports representing 19 services, and five LMP/CPVI reports representing 16 services. Their report-code distributions were, respectively, 172 Enabling/360 Restrictive/283 Mixed/115 Not reported; 118/171/186/114; and 44/29/47/35. These service-group summaries are exploratory descriptive patterns only: the LMP/CPVI group contains few report units and several services represented within cluster documents, and no service-type effect or ranking is inferred. Across groups, the dominant cross-case feature was coexistence rather than a simple division between supportive and restrictive services. Individualized activities, positive relationships, or family contact could appear in the same report as blanket movement restrictions, substituted decision-making, weak vocational opportunity, or failures to implement formal plans.

### 3.2 Formal participation frequently exceeded usable agency

Formal mechanisms of participation were common, but the reports distinguished their administrative presence from practical usability. Rights information, complaint procedures, signatures, guardianship documents, and individual plans were sometimes present without equivalent evidence that residents understood alternatives, participated in consequential decisions, or could refuse proposed arrangements. For example, C35 documented a formal decision-support procedure, but staff stated that beneficiaries could not understand the information provided and no information sessions were recorded (p. 10), whereas C12 documented information, counseling, dialogue, role play, simulation, and situational analysis used to support decisions (p. 11). Across the full corpus, supported decision-making/consent (A2b) was classified as 11 Enabling, 20 Restrictive, and 23 Mixed, with no Not reported cases.

Complaint systems showed the same distinction. C01 contained a complaints box, register, and formal procedure, but no entries were identified, so actual use or resident influence was not demonstrated (pp. 10–11); this pattern was coded Mixed rather than Enabling. Stronger agency evidence required documented use, response, participation, or influence rather than the presence of a procedure alone. Across codes, the analysis therefore treated administrative compliance, comprehension, and resident influence as separate evidentiary questions.

### 3.3 Freedom of movement was the clearest structural discriminator

Freedom of movement was the most consistent structural distinction between enabling and containing configurations. At code level, freedom of movement/self-directed mobility (A4) was Enabling in six reports, Restrictive in 30, Mixed in 14, and Not reported in four; independent community mobility (D2) showed the same six Enabling cases, 32 Restrictive, 12 Mixed, and four Not reported. C12 documented that residents could move locally independently or choose accompaniment (pp. 8, 12), while C35 described permanently closed gates and community access only with staff accompaniment (pp. 11–12). The contrast concerned resident control and individualized support, not merely whether outings occurred.

Organized outings did not offset blanket movement controls. C01 documented museums, cinema, shopping centers, seasonal events, and trips (pp. 18–19), yet local walks and outings required accompaniment (p. 15). Similar combinations appeared across the corpus: organized exposure could be extensive while resident-initiated mobility remained unavailable. This repeated within-report contrast is why organized community exposure and independent mobility were coded separately.

### 3.4 Community location did not guarantee community membership

The analysis separated availability of ordinary community resources (D1), independent use and mobility (D2), organized community exposure (D3), and sustained community membership or valued roles (D4). Resource access was Enabling in 26 reports and organized exposure in 32, whereas community membership/valued roles was Enabling in only six and Not reported in 33. The different coverage and directions show that proximity and supervised presence were much more commonly documented than reciprocal membership.

Concrete cases made this gradient visible. C24 documented recurring remunerated activities in local households and private space for resident couples (pp. 7, 9), evidence of recurring productive and relationship roles rather than one-off exposure. By contrast, reports such as C01 documented diverse community outings while independent movement remained restricted. The analytical distinction was therefore between being taken into community settings and holding resident-initiated, recurring, or reciprocal positions within ordinary community life.

### 3.5 Capability development was concentrated in daily living rather than education and work

Capability-development evidence was strongest for daily living. Independent-living skills (C1) were Enabling in 29 reports, Restrictive in eight, Mixed in 12, and Not reported in five. Cooking, cleaning, self-care, shopping, budgeting, occupational activity, and therapeutic programmes were recurrent, but these activities were not consistently connected to education, work, or broader adult roles.

Education and employment were substantially more restrictive. Education/qualification (C3) was Restrictive in 42 reports and Enabling in two; employment/valued productive roles (C4) was Restrictive in 37 and Enabling in two. C45 documented one resident attending ninth grade online together with writing and numeracy activities available to other interested residents (pp. 11–12), and C31 documented efforts to identify employment opportunities and short work placements of up to two months, although maintaining employment remained difficult (pp. 32–33). Conversely, C12 stated that beneficiaries were not eligible for work-capacity expertise because of chronic illnesses and that all had been considered unable to work following invalidity decisions (p. 24). These cases show why the analysis separated internal activity provision from opportunities that broaden future educational or productive roles.

### 3.6 Relational warmth and structural enablement were distinct dimensions

Positive staff-resident relationships were among the most frequently documented enabling features: staff-resident relational climate (B3) was Enabling in 33 reports, Restrictive in three, Mixed in 14, and Not reported in four. The same reports could nevertheless contain structural restrictions. In C01, the monitoring report described respectful, reciprocal, family-like everyday interactions between residents and staff (p. 28) while also documenting that local walks and outings required accompaniment (p. 15).

Accordingly, relational climate and structural opportunity were retained as separate dimensions of the report profile. A positive relational code did not override restrictive evidence concerning movement, surveillance, legal substitution, money control, or access to education and employment; equally, safeguarding concerns were coded only where the report supplied specific evidence. The resulting Mixed and cross-code patterns preserve within-report contradiction rather than forcing a global service judgment.

### 3.7 The documentation–practice gap was a cross-cutting institutional mechanism

The documentation-practice gap (E8) was coded Restrictive in 29 reports, Mixed in 21, Not reported in four, and Enabling in none. Person-centered planning/goal co-construction (C2) was Enabling in 12 reports, Restrictive in 20, and Mixed in 22. Reports repeatedly described plans, procedures, schedules, consent forms, or risk-management arrangements that were incomplete, generic, unsigned, retrospective, or weakly connected to documented implementation.

For example, C18 juxtaposed an extensive plan-based activity framework with monitoring observations of limited recreational participation and resident passivity (pp. 11–12), while C33 recorded plans and schedules that were present but not implemented (pp. 7, 10). These patterns were treated as implementation evidence rather than as proof of residents' subjective experience. Administrative completeness and resident-usable opportunity therefore remained analytically distinct.

### 3.8 Transition was uncommon, and administrative relocation was not equivalent to deinstitutionalization

Transition and deinstitutionalization pathways (D5) were Enabling in five reports, Restrictive in 34, Mixed in 14, and Not reported in one. Stronger evidence required more than transfer: the reports had to document resident preference or participation, preparation, a credible community-living pathway, and/or continuity after exit. C31 documented one successful integration in which a beneficiary was transferred initially to professional personal assistance, subsequently obtained employment, and lived independently (p. 26), providing a concrete resident-level pathway beyond organizational restructuring.

Other reports documented relocation without comparable evidence of resident-requested transition or reintegration planning. C26 documented that all 29 residents had been transferred from another residential service, while no request for transfer to the new service was recorded and beneficiary files lacked documentation of deinstitutionalization or family-reintegration steps (p. 18). Such evidence was classified as restrictive transition/relocation rather than as resident-led deinstitutionalization. A transfer record was therefore not treated as sufficient evidence of supported choice when the same documentary record did not demonstrate a resident request or documented deinstitutionalization/reintegration planning.

### 3.9 Configuration patterns and analytically informative cases

Cross-case comparison was used to organize reports into descriptive configuration families, not to score services. Of the 54 reports, 19 were summarized as enabling/community-bridging, six as activity-based/mixed, 16 as activity-based/containing, and nine as containing/critical; four thematic reports were left uncategorized because their restricted monitoring scope did not support a whole-profile assignment. Enabling/community-bridging profiles combined some resident control and mobility with individualized support and practical community connections; activity-based/containing profiles could show extensive programmes and positive relationships while retaining blanket restrictions; containing/critical profiles combined several restrictive mechanisms.

Boundary cases were analytically important because they prevented the families from becoming global quality labels. C12, for example, contained enabling evidence on supported decision-making and independent local mobility (pp. 8, 11–12) while also documenting restrictive vocational expectations (p. 24). C01 combined a positive staff-resident climate (p. 28) with accompaniment-based mobility (p. 15). Across cases, these contradictions were retained in the report profile and analytic memo; the family label summarized the configuration but did not erase countervailing evidence.

### 3.10 Sensitivity analysis

Sensitivity analyses tested whether the principal patterns depended on multi-service cluster reports or thematic reports. Excluding the four cluster reports yielded 50 reports representing 50 services and 1,317 documented directional decisions; excluding the four thematic reports yielded 50 reports representing 58 services and 1,369 documented decisions; excluding both yielded the most conservative set of 47 reports/47 services and 1,287 documented decisions. Key restrictive counts changed little for movement (A4: 30 in the primary analysis, 29 with clusters excluded, 30 with thematic reports excluded, and 29 with both excluded), education (C3: 42/39/42/39), employment (C4: 37/34/37/34), and the documentation-practice gap (E8: 29/25/25/22). The main distinctions–formal procedure vs. usable agency, organized exposure vs. independent participation, relational warmth vs. structural autonomy, and administrative relocation vs. supported transition–therefore remained visible in the conservative scenario. Complete sensitivity outputs are provided in Supplementary Data 1, and Table 3 summarizes the cross-case mechanisms carried forward into the Discussion.

**Table 3:** Final cross-case mechanisms for the results and discussion transition.

Mechanism	Documentary pattern	Interpretive implication	Status after full corpus
Formal procedure vs. usable agency	Procedures, signatures and plans often exceed evidence of comprehension or influence.	Administrative compliance should not be equated with autonomy.	Stable
Freedom of movement	Independent movement differentiates enabling cases; blanket accompaniment and locked access characterize containing cases.	Mobility is a pivotal psychosocial mechanism.	Stable
Exposure vs. membership	Community outings are commoner than independent use, reciprocal ties or valued roles.	Community inclusion requires more than physical presence.	Stable
Activity volume vs. enablement	High activity density may remain staff-directed and internally focused.	Programme quantity is a weak proxy for agency or flourishing.	Stable
Relational warmth vs. structure	Positive staff relationships coexist with surveillance, guardianship and movement control.	Relational and structural dimensions must be analyzed separately.	Stable
Plan vs. practice	Formal plans and procedures are often incomplete, generic or weakly implemented.	Implementation is the mechanism connecting person-centered policy to opportunity.	Stable
Small scale	Small settings can support household agency but may retain institutional controls.	Scale is a boundary condition, not a sufficient cause.	Stable
Transition vs. relocation	Resident-led preparation and continuity differ from administrative transfer.	Deinstitutionalization should be defined by supported choice and sustained community living.	Stable
Low expectations	Generalized incapacity narratives narrow education, work and risk-taking opportunities.	Staff expectations shape capability conversion.	Stable
Reciprocal roles	Employment, paid tasks, couples and sustained external roles are rare but differentiating.	Valued roles are high-information qualitative indicators of enablement.	Stable

## 4 Discussion

### 4.1 Residential services are meaningful psychosocial contexts

The central finding is that residential service models do not determine psychosocial enablement by themselves. Across the complete corpus, the same formal service categories contained markedly different combinations of autonomy, relationship support, capability development, mobility, and community connection. The explanatory unit is therefore not the service label alone but the configuration of everyday rules, staff practices, legal arrangements, environmental affordances, implementation capacity, and community bridges. Residential services can consequently be understood as meaningful organizational and social contexts whose structures expand or narrow documented opportunities relevant to agency, belonging, inclusive participation, and quality of life.

This interpretation is compatible with organizational research in intellectual-disability services. Staff outcomes vary by residential model and organizational characteristics (Chou et al., 2010; Thompson and Rose, 2011), while resident and professional perspectives indicate that organizational resources, shared values, stability, and the living environment shape the quality of support (Olivier-Pijpers et al., 2019, 2020). Our documentary findings extend this literature by specifying documented mechanisms that shape resident opportunity: mobility rules, comprehensible information, control of money, relationship privacy, person-centered implementation, staff expectations, and conversion of nearby community resources into ordinary social participation.

### 4.2 Freedom of movement links autonomy to dignity of risk

Freedom of movement was the clearest structural discriminator between enabling and containing configurations. This is theoretically important because mobility is both an expression of autonomy and a precondition for many other opportunities. A resident who cannot leave without permission or universal accompaniment may formally have access to shops, family, worship, leisure, education, or employment while being unable to convert that access into self-directed action. In SDT terms, the environment constrains autonomy; in capability terms, a nominal resource is present but substantive freedom is reduced (Ryan and Deci, 2000; Terzi, 2020).

The finding also clarifies the difference between individualized support and institutional risk control. Some residents required assistance for legitimate reasons, but blanket rules applied to everyone were analytically different from proportionate, person-specific support. The dignity-of-risk literature explicitly warns that benevolent protection can become paternalistic when it removes the person's opportunity to decide, learn, and participate (Chicoine and Kirschner, 2022). Locked gates, universal escort rules, and permission systems should therefore not be interpreted only as security procedures; in the monitoring record, they also narrowed the range of resident-controlled opportunities.

### 4.3 Formal rights and documents were weaker than usable agency

A second major finding was the recurrent distance between formal participation and usable agency. Rights information, complaint boxes, consent forms, guardianship records, and individual plans were common, but the reports often provided weaker evidence that residents understood these mechanisms, could refuse, could compare alternatives, or could influence outcomes. The distinction is central to both supported decision-making and the capability approach. Administrative form cannot substitute for the resident's ability to exercise a choice. A signature obtained without demonstrated comprehension, or a plan prepared without resident participation, represents documentation rather than substantive agency.

This result is especially relevant to transfers. Several reports documented relocations in which evaluation, consent, financial records, or continuity of care were weak or *post hoc*. Such moves may reduce or alter institutional placement, but they do not meet a psychosocial definition of deinstitutionalization if the person has not participated meaningfully in the decision and transition. Article 19 of the Convention on the Rights of Persons with Disabilities provides a secondary normative reference point because it links community living with choice of residence, inclusion, and access to ordinary community services (United Nations, 2006). Our analysis does not convert this legal standard into a psychological outcome measure; rather, it shows that supported choice and practical inclusion are also central to the psychological constructs of autonomy and agency.

### 4.4 Activity provision should be separated from competence and valued roles

The results also challenge the assumption that a dense activity programme is evidence of enablement. Many services offered crafts, games, occupational activities, celebrations, religious events, and excursions. These activities may be enjoyable and relationally valuable, but they do not necessarily develop competence, expand future options, or transfer control to residents. This distinction is consistent with active-support research, which treats engagement in meaningful activity as an outcome requiring individualized support rather than as the mere presence of scheduled occupation (Mansell et al., 2002). The most striking capability gap concerned education and employment: restrictive evidence greatly outnumbered enabling evidence, and generalized assumptions about age, diagnosis, disability severity, or work capacity sometimes narrowed expectations before individualized assessment occurred.

The contrast is consistent with SDT's competence dimension and with research on person-centered planning. Ratti et al. (2016)) found that person-centered planning can improve daily choice and community participation, but broader outcomes such as employment remain inconsistent. (Mansell and Beadle-Brown 2004) similarly argued that planning must become action through changes in power, resources, staff practice, and organizational arrangements. The present corpus provides a documentary analog of that argument: individualized plans were most meaningful when linked to observable skill development, resident-defined goals, work, household responsibility, mobility, or transition.

### 4.5 Relational warmth is necessary but not sufficient for belonging

Positive staff-resident relationships were common even in structurally restrictive services. This is an important negative-case pattern. Respectful, warm staff interactions may support opportunities for relatedness, but the reports did not establish residents' subjective emotional security, and such interactions cannot by themselves remove guardianship, surveillance, inaccessible space, or blanket mobility restrictions. Conversely, structural reforms without trustworthy relationships may also fail to support belonging. The finding therefore supports treating relational climate and structural opportunity as distinct but interacting layers of the residential environment.

Documentary evidence relevant to belonging was most enabling when relationships extended beyond staff support to family continuity, reciprocal peer or couple relationships, private communication, and roles recognized outside the service. This distinction helps explain why reciprocal community roles were analytically more informative than one-off outings. Employment, paid community tasks, stable external relationships, volunteering, and supported couple life represent social positions in which residents can be contributors and relationship partners rather than only recipients of services. Work on valued community roles has similarly emphasized their importance for building social relationships (Harlan-Simmons et al., 2001).

### 4.6 Community living depends on conversion, not location alone

The findings align with international evidence that community living is generally associated with greater quality of life, freedom, self-determination, relationships, and community activity, while outcomes remain heterogeneous (Walsh et al., 2010; McCarron et al., 2019). Recent longitudinal evidence is especially consistent with the present mechanism-based interpretation: Navas et al. (2025)) found that moving to a community setting was not sufficient by itself; improvements in quality of life depended on continuing opportunities for decision-making and control. Qualitative research on post-care transitions among people with mild intellectual disabilities similarly describes sustainable adulthood as supported interdependence rather than detachment from support, emphasizing relational continuity and practical assistance with decision-making, employment, finances, and service navigation (Tapan et al., 2026). Our analysis identifies documentary conditions that can enable or block this conversion. Proximity to transport, shops, cultural venues, or family did not demonstrate usable opportunity when the same reports documented locked gates, universal staff accompaniment, inaccessible money, or plans that did not support resident-initiated use.

The three Ceua su de Câmpie services are particularly informative because small scale and shared geography did not erase differences in relationship autonomy, remunerated roles, movement rules, and family continuity. Similar contrasts appeared across services sharing provider traditions or localities. This supports treating scale and service type as contextual descriptors rather than as sufficient indicators of psychosocial opportunity. Community living is better conceptualized as an achieved pattern of agency, ordinary participation, reciprocal relationships, and control than as a postal address or administrative category.

### 4.7 The documentation-practice gap is an organizational implementation mechanism

The documentation-practice gap connects the study's psychological and organizational findings. Procedures and plans can express progressive values while everyday implementation remains limited by vacancies, shared specialists, weak leadership, inaccessible environments, or habitual institutional routines. Research on organizational readiness for change in disability healthcare emphasizes that resources and organizational dynamics affect implementation capacity (Granberg et al., 2025). The current findings suggest that implementation capacity is also relevant to psychosocial opportunity: when a resident-defined goal remained only in documentation, the report did not demonstrate its conversion into practice.

This helps explain why services with apparently similar formal requirements produced different configurations. The issue is not simply whether the organization possesses a policy, but whether staffing, professional expectations, decision support, physical space, and community partnerships allow that policy to become ordinary practice. The result also cautions against using documentary completeness as a proxy for wellbeing or quality of life.

### 4.8 Contribution to positive psychology: from individual resources to opportunity structures

The study contributes to positive psychology by treating wellbeing-relevant conditions as context dependent. It does not measure happiness, life satisfaction, or subjective wellbeing and therefore cannot claim that a particular service caused residents to flourish. Instead, it identifies organizational conditions theoretically linked to autonomy, competence, relatedness, agency, belonging, participation, and the possibility of valued social contribution. This responds to a contextual challenge for positive psychology: strengths and psychological resources can be difficult to exercise when the surrounding opportunity structure restricts movement, information, decision authority, relationships, learning, or access to ordinary social roles.

The combined SDT-capability interpretation provides a useful conceptual bridge. SDT identifies psychologically important needs; the capability approach asks whether the person has real freedom to pursue valued ways of living; and the documentary analysis identifies the organizational mechanisms that expand or narrow those freedoms. Within this framework, locked movement, inaccessible information, substituted decisions, weak privacy, absent education, and low vocational expectations are not merely service deficiencies. They are contextual constraints on the conditions under which autonomy, competence, relationships, and social contribution can develop.

### 4.9 Practice and policy implications

Several implications follow. Monitoring and service development should distinguish organized activity from resident-chosen meaningful activity; community exposure from independent participation; proximity from actual use; signed documentation from supported understanding; and transfer from resident-led transition. Services seeking to become more enabling should prioritize individualized mobility support, accessible decision support, resident control of everyday money and possessions, privacy and relationship autonomy, implementation of resident-defined plans, educational and vocational pathways, and partnerships that generate recurring community roles rather than episodic outings. These priorities also shift practice toward co-construction: supportive environments are not defined only by what organizations provide, but by whether residents can influence and use those resources in ways connected to their own goals.

Organizational change should also target the mechanisms that make restrictive practice self-reinforcing: staff shortages, generalized risk rules, low expectations, clinical dominance, inaccessible environments, and administrative routines. Small-scale relocation without changes in these mechanisms may reproduce institutional life in a different building. Conversely, even larger services may improve psychosocial opportunities when they replace blanket rules with individualized support and create credible pathways into ordinary community roles.

### 4.10 Strengths and limitations

A major strength is the complete eligible documentary corpus from the national authority's 2025 archive under the stated eligibility rules, combined with one stable 31-code framework, report-level audit trails, original-page verification of high-impact findings, exhaustive second-pass review, negative-case analysis, and sensitivity analyses for cluster and thematic reports. The use of Not reported also reduced the risk of treating documentary silence as evidence of absence.

The limitations are equally important. Monitoring reports were produced for statutory oversight rather than research and vary in length, visit focus, evidence density, and reporting style. They are not neutral mirrors of practice: what becomes visible is shaped by monitoring mandates, inspection priorities, professional language, documentary requirements, and what staff, residents, records, and observations made available during the visit. Accordingly, Not reported denotes documentary silence within that monitoring context and cannot establish absence of a practice. Residents were not recruited or interviewed by the author, so the analysis cannot substitute for first-person accounts of wellbeing, satisfaction, identity, or perceived autonomy. Directional codes are analytic summaries rather than validated scales, and counts should not be interpreted as service scores or outcome prevalence. The report-level unit also involves a tradeoff: treating four multi-service reports as single units prevents inflation of shared findings but may conceal within-report variation between individual services when the source does not permit separate attribution. The sensitivity analyses address the influence of these clusters but cannot recover information not differentiated in the original documents. Finally, the single-analyst, AI-assisted workflow provides consistency, explicit source tracing, and a detailed audit trail but does not provide independent human intercoder agreement. The study concerns the Romanian monitoring context and should be generalized analytically through mechanisms rather than statistically to other national systems.

## 5 Conclusion

Across 54 monitoring reports representing 67 residential services, psychosocial opportunity was shaped less by service labels than by the interaction of everyday choice, supported understanding, movement rules, relationship support, staff expectations, plan implementation, environmental accessibility, and effective connections with ordinary community life. The most enabling configurations converted organizational resources into resident-controlled opportunities: movement, relationships, learning, household agency, productive roles, and supported transition. The most containing configurations retained formal procedures and activities while narrowing the resident's practical control over when, where, with whom, and for what purposes daily life could occur.

The findings therefore support a contextual account of wellbeing in residential disability services. Autonomy, competence, relatedness, and community inclusion cannot be understood only as individual attributes; their exercise also depends on whether institutional environments provide usable opportunities for valued ways of living. These findings concern documented wellbeing-relevant opportunity structures, not measured wellbeing outcomes or residents' subjective experiences. For positive psychology, meaningful contexts should therefore be evaluated not only by the resources they contain, but by whether organizational arrangements convert those resources into substantive opportunities for people to understand, choose, relate, contribute, participate, and pursue valued life directions.

## Data Availability

The original contributions presented in the study are included in the article/Supplementary material, further inquiries can be directed to the corresponding author.
